# Improvement of Commercially Valuable Traits of Industrial Crops by Application of Carbon-based Nanomaterials

**DOI:** 10.1038/s41598-019-55903-3

**Published:** 2019-12-18

**Authors:** Kamal Pandey, Muhammad Anas, Victoria K. Hicks, Micah J. Green, Mariya V. Khodakovskaya

**Affiliations:** 1 Department of Biology, University of Arkansas at Little Rock, Little Rock, AR 72204 USA; 2 Artie McFerrin Department of Chemical Engineering, Texas A&M University, College Station, TX 77843 USA

**Keywords:** Nanoparticles, Abiotic

## Abstract

Carbon-based nanomaterials (CBNs) have great potential as a powerful tool to improve plant productivity. Here, we investigated the biological effects of graphene and carbon nanotubes (CNTs) on fiber-producing species (cotton, *Gossypium hirsutum*) and ornamental species (vinca, *Catharanthus roseus*). The exposure of seeds to CNTs or graphene led to the activation of early seed germination in *Catharanthus* and overall higher germination in cotton and *Catharanthus* seeds. The application of CBNs resulted in higher root and shoot growth of young seedlings of both tested species. Cultivation of *Catharanthus* plants in soil supplemented with CBNs resulted in the stimulation of plant reproductive system by inducing early flower development along with higher flower production. *Catharanthus* plants cultivated in CNTs or graphene supplemented soil accelerated total flower production by 37 and 58%, respectively. Additionally, CBNs reduced the toxic effects caused by NaCl. Long-term application of CBNs to crops cultivated under salt stress conditions improved the desired phenotypical traits of *Catharanthus* (higher flower number and leaf number) and cotton (increased fiber biomass) compared to untreated plants of both species cultivated at the same stress condition. The drought stress experiments revealed that introduction of CBNs to matured *Catharanthus* plant increased the plant survival with no symptoms of leaf wilting as compared to untreated *Catharanthus* growing in water deficit conditions.

## Introduction

The applications of nanotechnology in agriculture can lead to significant improvements in plant growth and yield and reduce the use of genetically modified organisms (GMO) and agrochemicals^1^. In the last decade, studies on plant-nanomaterial interactions have increased rapidly due to the discovery of the positive effects of nanomaterials *in planta*^2–4^. The applications of nanomaterials in plant agriculture are widespread, ranging from nano-fertilizers for improving growth and yield, pesticides for controlling pest and plant disease, and nanosensors for managing plant health and soil quality^5^. It is reported that carbon-based nanomaterials (CBNs) cover around 40% of all engineered nanomaterials used for agricultural applications^6^. To date, the interactions of CBNs with plant systems have been studied in different plant organs including seeds, seedlings, plants, and plant cell cultures^6–17^. Positive effects of CBNs on seed germination and plant productivity have been documented for model plant species^8,11–13^, food crops (corn, barley, soybean)^14^ as well as for bioenergy crops (sorghum, switchgrass)^15^. One of the most promising discoveries is the ability of a wide range of CBNs to induce early flower development and significantly stimulate the production of flowers and fruits of plants grown in hydroponics or soil supplemented with CBNs^16,17^. This discovery may lead to the establishment of highly efficient technology with a focus on the improvement of commercially important crops such as flower producing species (ornamental plants) or/and fiber producing plants (cotton).

However, the mechanism of positive effects of CBNs on plant systems is not fully understood yet. It is already known that CBNs are affecting plants at the molecular level during exposure because it has been determined that the positive impact of CBNs on seed germination and plant growth is strongly correlated with the regulation of expression of major stress-responsive genes in CBN-exposed plants^8–13^. For example, the exposure of plants to CNTs led to the activation of the expression of aquaporins (water channel genes) in different crop species^8,11,12,14,15^. Many recent efforts were focused on detection of CBNs in plants. It was shown that CBNs used as growth regulators can be absorbed by roots of plants and move from roots to other plant organs including shoots, leaves, flowers, and fruits^8,11–15,17^. It was interesting that absorption of CBNs not only led to changes at the genomic level but also imposed changes in the metabolomic profile in CNT-treated plants^8,17^. The documented presence of absorbed CBNs in the edible plant organs (flowers and fruits) raised significant concerns about the possible movement of CBN residues located in plant organs into the food chain^4^. At the same time, the use of CBNs for the regulation of industrial plant species productivity is a wisely profitable approach since CBNs can simultaneously improve multiple commercially valuable traits (seed germination, biomass production, fruit/fiber bolls production) without transfer of CBNs to the potential consumer.

Recently, we demonstrated that CBNs not only improved plant productivity under normal conditions of cultivation but can also reverse certain symptoms of toxicity caused by abiotic stress^15^. For example, exposure of seeds or seedlings of bioenergy crops (sorghum and switchgrass) to CBNs can lead to a reduction of the toxic effects of NaCl (salt stress)^15^. Therefore, application of nanomaterials for enhancement of abiotic stress tolerance of commercially valuable crops is a highly favorable approach in current situation of global climate change. It is reported that environmental stress is becoming a crucial challenge for agricultural production^18–24^. The paucity of freshwater resources and the dramatically increased salinization is a critical challenge for plant yield due to rapid desertification of agricultural land^18,19^. It is expected that osmotic stresses such as drought and salt may cause serious salinization of more than 50% of arable lands throughout the world by the year 2050^20,21^. The excessive exposure of plants to salts lead to leaf wilting followed by reduced growth and eventually partial or whole plant damage^21,22^. It is reported that salt stress significantly affects cotton productivity by reducing the shoot growth, monopodia per plant, plant height, cotton boll number, biomass, and fiber quality^23^. Similarly, irrigation of salty water on important ornamental plants negatively affected plant architecture and eventually led to delays in flower bud production^24^. Genetic modifications such as genetic engineering and plant breeding are quite popular tools to improve commercially valuable traits of plants, including overall productivity and abiotic stress tolerance^25,26^. However, limited availability of genes of particular crops, low success rate, potential ecological impacts as well as public concern about the genetically modified crops are the major disadvantages of existing technologies^25–27^. It is possible that the use of CBNs can provide an alternative approach for regulation of stress tolerance of non-food crop species.

**Figure 1:**
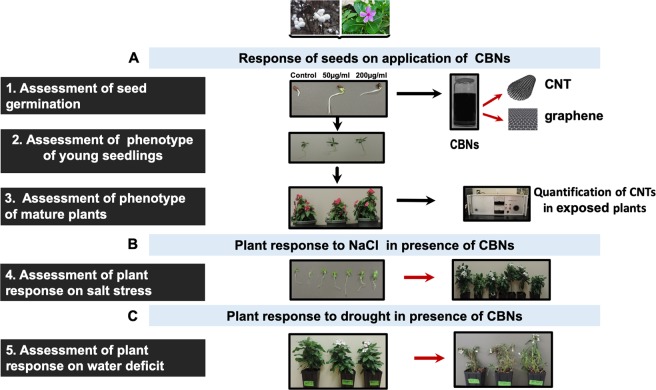
Experimental design for the study of the impact of CBNs on seed germination, plant growth, and osmotic stress tolerance of selected fiber-producing (cotton) and ornamental species (*Catharanthus*). The seeds of *Catharanthus* and cotton were exposed to growth media with CNTs or graphene. Long-term application of CBNs to tested crops was achieved by cultivating in the soil supplemented with CNTs or graphene.

The major goal of this work is to investigate the technological potential of two types of CBNs: multi-walled carbon nanotubes (CNTs) and graphene for the enhancement of productivity of ornamental plant (*Catharanthus*) and fiber-producing crop (cotton) under regular conditions and conditions with imposed drought and salt stresses. Figure 1 illustrates the experimental design of this study.

## Results

### Effects of CBNs on seed germination and growth of cotton and *Catharanthus* seedlings *in vitro*

**Figure 2:**
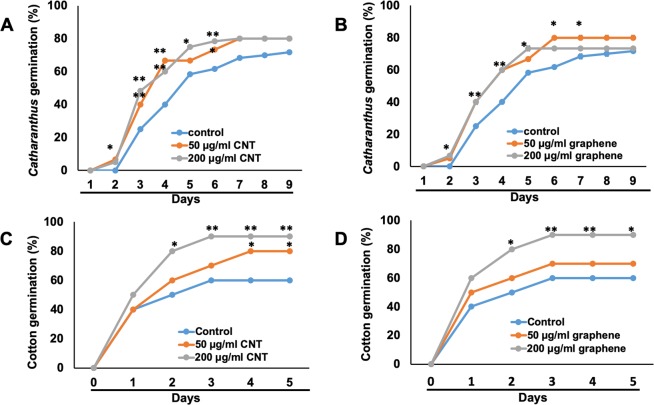
Enhancement of seed germination of fiber-producing and ornamental species by the application of CBNs. Effects of CNTs (**A**,**C**) and graphene (**B**,**D**) added to the growth medium on seed germination of *Catharanthus* (**A**,**B**) and cotton (**C**,**D**). The presented graph shows the percentage of seed germination by exposures to CBNs at a concentration of 50 µg/ml and 200 µg/ml. The entire seed germination experiments were repeated twice (n = 60 for each treatment and for conrol). The statistical significance was determined as compared to control (untreated) seeds by p < 0.05 and p < 0.01 (**p < 0.01 and *p < 0.05).

In order to test whether graphene and CNTs can affect the germination of two selected non-food species, sterilized seeds of *Catharanthus* and cotton were inoculated in growth medium supplemented with graphene or CNTs. Two different concentrations of CBNs (50 µg/ml and 200 µg/ml) were selected for germination tests and seedling development assay using data previously generated for other plant species^8,12^. We observed that the exposure of seeds to pure CBNs did not lead to the development of any toxic symptoms and positively affected the seed germination of cotton and *Catharanthus* (Fig. 2). The early germination was recorded as the result of the application of graphene or CNTs to seeds of *Catharanthus*. For example, the first signs of seed germination were observed at day 2 for seeds exposed to CBNs whereas germination was started at day 3 for *Catharanthus* seeds exposed to control medium which was not supplemented with CBNs. Moreover, both tested CBNs significantly increased the germination rate of *Catharanthus*. (Fig. 2A,B). For instance, the seeds exposed to CNTs and graphene increased germination rate by 27% and 20% respectively at day 4 as compared to untreated *Catharanthus*. The positive effects of CBNs on seed germination was observed for cotton as well. The cotton seeds exposed to 200 µg/ml CBNs germinated more effectively as compared to seeds exposed to 50 µg/ml CBNs (Fig. 2C,D). Thus, cotton seeds exposed to 50 µg/ml and 200 µg/ml CNTs increased germination by 20% and 30% respectively at day-4. Similarly, the application of 50 µg/ml and 200 µg/ml graphene to cotton seeds increased germination by 10% and 30% respectively at day 4.

**Figure 3:**
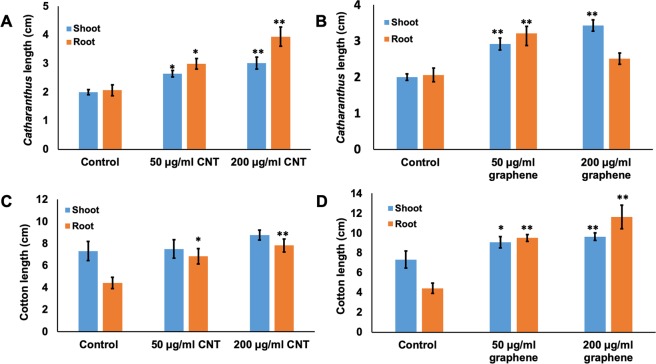
The activation of growth and development of seedlings of cotton and *Catharanthus* exposed to two types of CBNs. Effects of CNTs (**A**,**C**) and graphene (**B**,**D**) on the growth of seedlings of *Catharanthus* (**A**,**B**) and cotton (**C**,**D**). The presented graphs show the root length and shoot length of 4 week-old *Catharanthus* and 1 week-old cotton exposed to 50 µg/ml and 200 µg/ml CBNs. Experiments were repeated twice (n = 20 for each treatment and for control). The statistical significance was determined as compared to control (untreated) seedlings by p < 0.05 and p < 0.01 (*p < 0.05 and **p < 0.01).

The phenotypical analysis of germinated seedlings revealed that seedlings exposed to CBNs (CNTs, graphene) grew faster compared to untreated seedlings of both tested crops. Application of CBNs at both concentrations (50 µg/ml, 200 µg/ml of CNTs or graphene) significantly affected the root length as well as shoot length of 1 week-old cotton and 4 week-old *Catharanthus* (Fig. 3). The introduction of 200 µg/ml CNTs to 4 week-old *Catharanthus* seedlings increased the root length and shoot length by 91% and 51% respectively (Fig. 3A). Whereas, the application of 200 µg/ml graphene increased the *Catharanthus* shoot length by 72% and root length by 22% as compared to untreated *Catharanthus* seedlings (Fig. 3B). Similar results were observed for cotton seedling development by exposure to CBNs (Fig. 3C,D). In particular, root length of seedlings was significantly increased by the application of 50 µg/ml and 200 µg/ml CNTs or graphene and shoot length was significantly increased by the application of 200 µg/ml of graphene. Specifically, the exposure of cotton seedlings to 200 µg/ml CNTs accelerated the shoot length by 20% and root length by 76% (Fig. 3C). Whereas, the exposure of cotton seedlings to 50 µg/ml graphene accelerated the shoot length by 24% and root length by 115% as compared to control cotton seedlings (Fig. 3D).

**Figure 4:**
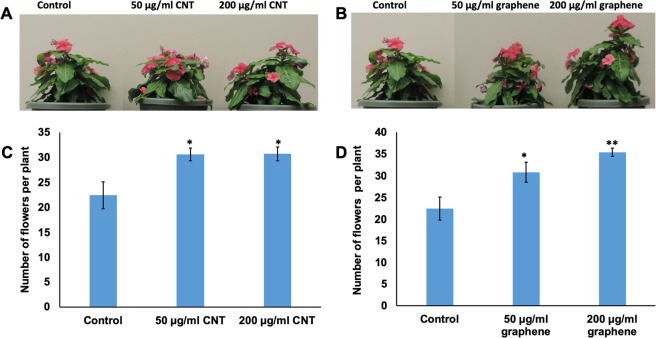
Activation of flower production in *Catharanthus* as a result of the application of CBNs (CNT, graphene) to the soil mix. Effects of CNTs (**A**,**C**) and graphene (**B**,**D**) on the total number of produced flowers. The statistical significance was determined as compared to untreated (control) *Catharanthus* plants. The entire plant growth and flower production experiments were repeated twice (n = 15 for each treatment of *Catharanthus*). The statistical significance was determined as compared to control (untreated) plants by p < 0.05 and p < 0.01(*p < 0.05 and **p < 0.01).

Moreover, we also investigated the effects of CBNs on biomass production of young tested crops and found that the introduction of graphene or CNTs to growth medium significantly increased the total biomass production (Supplementary Tables S1 and S2). The application of CBNs stimulated the fresh root as well as shoot biomass production in 4 week-old *Catharanthus*. For example, seedlings exposed to 50 µg/ml CNTs accelerated fresh shoot biomass production by 33%, whereas seedlings exposed to 200 µg/ml graphene accelerated the fresh shoot biomass production by 53% (Supplementary Table S1). We also observed the significant effects of CBNs on fresh biomass yield in the fiber producing plant such as cotton (Supplementary Table S2). Based on the phenotypic analysis of young seedlings (*Catharanthu*s, cotton), we have concluded that the application of low doses of CBNs can positively affect the overall growth pattern of model ornamental crops (*Catharanthus*) and valuable fiber producing crops (cotton) at the seedlings stage without showing any toxic symptoms. In addition, we also investigated whether the continuous exposure of both crops to carbon-based nanomaterials can induce any desired traits in cotton and *Catharanthus* at the stage of maturity. We observed that the introduction of CBNs to soil used for the cultivation of *Catharanthus* dramatically affected the reproductive system of both tested species. For example, *Catharanthus* plants exposed to CBNs (graphene, CNTs) resulted in early flower development (Supplementary Fig. S1A,B) as well as significantly increased the number of flowers as compared to untreated *Catharanthus* plants (Fig. 4). Specifically, *Catharanthus* plants exposed to 50 and 200 µg/ml CNTs increased the total number of flowers by 37%. Similarly, *Catharanthus* exposed to 50 and 200 µg/ml graphene increased the total number of flowers by 37% and 58%, respectively at 20 week-old plants (Fig. 4). We found that addition of CBNs to the cotton cultivated in the soil led to early flower development as compared to untreated cotton at 10 week-old plants (Fig. S1C,D). However, the number of produced fiber bolls in CBN-treated and untreated (control) cotton plants were not visibly different (Fig. S2).

### Effect of CBNs on seed germination of *Catharanthus* and cotton exposed to salt stress

**Figure 5:**
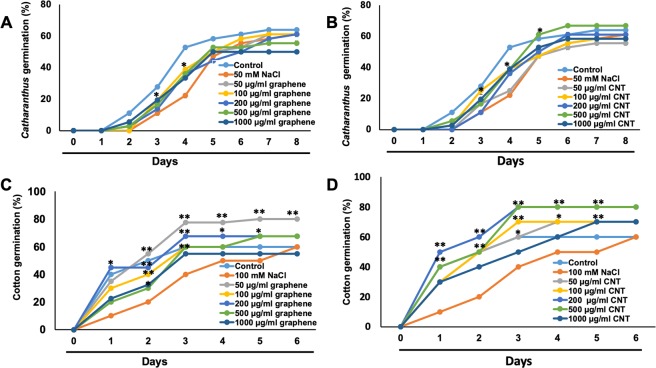
Activation of seed germination by application of CBNs in cotton and *Catharanthus* under salt stress. Effects of graphene (**A**,**C**) and CNTs (**B**,**D**) on seed germination of *Catharanthus* (**A**,**B**) and cotton (**C**,**D**) exposed to salty growth media. 50 mM NaCl and 100 mM NaCl were used to impose salt stress in *Catharanthu*s and cotton, respectively. The seed germination experiments were repeated twice (n = 60 for each treatment of cotton and *Catharanthus*) The statistical significance was determined as compared to seeds exposed to only NaCl by p < 0.05 and p < 0.01 (*p < 0.05 and**p < 0.01).

Sodium chloride (NaCl) is toxic for both tested species and reduces the rate of germination of *Catharanthus* and cotton. We tested the effect of different concentrations of NaCl on seed germination of both tested crops (Supplementary Fig. S3). The addition of 50 mM NaCl to the growth medium reduced the *Catharanthus* germination by 25% at day 3 as compared to untreated (control) *Catharanthus* seeds (Supplementary Fig. S3A). Similarly, the addition of 100 mM NaCl to growth media reduced cotton germination by 10% at day 4 as compared to untreated (control) cotton seeds (Supplementary Fig. S3B). Based on these observations, we selected 50 mM and 100 mM of NaCl for germination tests involving seeds of *Catharanthus* and cotton, respectively, in conditions of CBN-treatment (Fig. 5). The application of CBNs to salty growth medium reversed the toxic effects of salts and positively affected the seed germination of both tested crops (Fig. 5). The CBNs concentrations at a range of 100 µg/ml to 200 µg/ml were the most effective for reversal of inhibition of *Catharanthus* seed germination caused by salt stress (Fig. 5A,B). Both tested nanomaterials (CNTs, graphene) significantly activated the cotton germination as well (Fig. 5C,D). For example, the application of 50 and 100 µg/ml CNTs to NaCl exposed cotton seeds resulted in a 20% increase in germination as compared to cotton seeds treated with only NaCl at day-4. The addition of 50 µg/ml graphene to NaCl exposed seeds increased the cotton germination by 28% as compared to cotton seeds treated with only NaCl (Fig. 5C,D). On the other hand, the addition of activated carbon (micro-sized carbon particles) to the 50 mM NaCl supplemented growth medium did not lead to improvement of *Catharanthus* seed germination affected by the salt stress (Supplementary Fig. S5A).

### Effects of CBNs on growth and yield of cotton and *Catharanthus* exposed to salt stress

**Figure 6:**
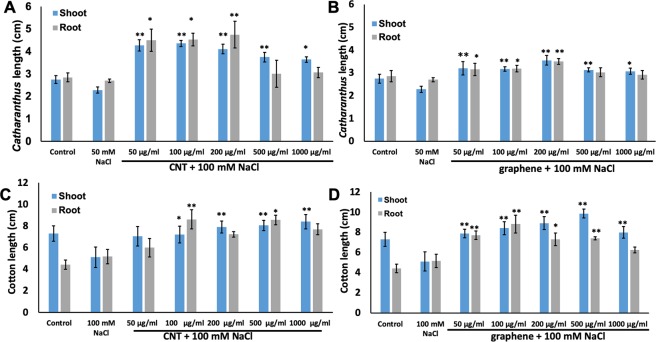
Growth and developments of seedlings of cotton and *Cathatanthus* exposed to CBNs under salt stress *in vitro*. Effects of CNTs (**A**,**C**) and graphene (**B**,**D**) on the growth of 4-week-old *Catharanthus* (**A**,**B**) and 1-week-old cotton (**C**,**D**) exposed to agar MS medium supplemented with NaCl. The seedlings development experiments were repeated twice (n = 20 for each treatment of cotton and *Catharanthus*). The statistical significance was determined as compared to seedlings treated with only NaCl by p < 0.05 and p < 0.01 (*p < 0.05 and **p < 0.01).

In order to investigate the effect of two CBNs on tolerance of young and mature cotton and *Catharanthus* plants to salt stress, we performed several experiments. First, we tested the sensitivity of *Catharanthus* and cotton seedlings to different doses of NaCl (Supplementary Fig. S4). Based on the observed intensity of toxicity of NaCl in tested species, we selected 50 mM NaCl for *Catharanthus* and 100 mM NaCl for cotton in further stress experiments. As shown in Fig. 6A,B, 4-week-old *Catharanthus* seedlings exposed to 50 mM NaCl reduced the root length by 5% and shoot length by 17% as compared to control (untreated) seedlings. However, the phenotypic analysis of young seedlings exposed to both CBNs and NaCl revealed that the introduction of both tested CBNs to salty growth medium dramatically reduced the toxic symptoms of NaCl and positively affected the seedling growth of both *Catharanthus* and cotton (Fig. 6). In fact, exposure of young seedlings (cotton, *Catharanthus*) to salty growth media resulted in higher root and shoot length as compared to control seedlings (seedlings grown in regular growth media). Specifically, CBN concentrations of 50–200 µg/ml resulted in better seedlings developments as compared to that of untreated seedlings in *Catharanthus*. For example, the introduction of 100 µg/ml CNTs to the salty medium (50 mM NaCl) increased shoot length by 91% and root length by 68% as compared to *Catharanthus* seedlings exposed to NaCl only (Fig. 6A). Similarly, the application of 100 µg/ml graphene led to in increase in root length by 18% and shoot length by 89%, as compared to *Catharanthus* seedlings exposed to NaCl only (Fig. 6B).

For the cotton, we found that the addition of 100 mM NaCl to the agar growth medium reduced the shoot length by 30% of young cotton plants **(**Fig. 6C,D; Supplementary Fig. S4B). At the same time, the application of CBNs to salty agar medium resulted in the reversal of NaCl toxicity and improvement of seedlings development towards normal (control) level. For example, the introduction of 1000 µg/ml CNTs to NaCl supplemented growth medium resulted in an increase of cotton shoot length by 64% as compared to seedlings exposed to medium supplemented with only NaCl. Similarly, the addition of 100 µg/ml CNTs resulted in an increase in root length by 67% as compared to cotton seedlings exposed to NaCl only (Fig. 6C). We observed similar effect of graphene on the development of cotton seedlings under salt stress conditions. For example, the application of 500 µg/ml graphene led to increase in shoot length by 93% as compared to seedlings exposed to NaCl only. Similarly, the introduction of 100 µg/ml graphene showed an increase in root length by 71% compared to seedlings exposed to NaCl only (Fig. 6D). The applications of CNTs and graphene improved the overall root and shoot biomass of *Catharanthus* seedlings grown under salt stress (Supplementary Fig. 6A,B). Note that the application of activated carbon (micro-sized carbon particles) to salty growth medium did not lead to suppression of toxic symptoms caused by NaCl observed for CNTs and graphene (Supplementary Fig. S5B,C).

**Figure 7:**
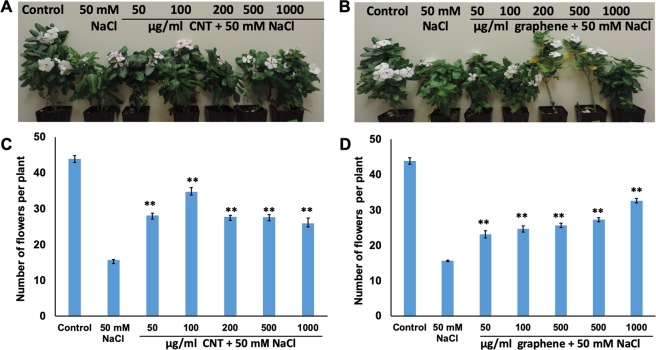
Long-term application of CBNs to salty soil reduced the toxic effects of salt stress and improved the growth and yield of *Catharanthus*. The introduction of CBNs to salty soil positively affected the production of flowers in *Catharanthus* cultivated in CNT-mixed soil (**A**,**C**) and graphene mixed-soil (**B**,**D**) under imposed salt stress. Control *Catharanthus* were grown in regular soil, NaCl exposed *Catharanthus* were grown at soil supplemented with 50 mM NaCl and CBNs exposed *Catharanthus* were cultivated in soil supplemented with 50 mM NaCl in presence of different concentrations of CNTs or graphene. The greenhouse experiment was repeated twice (n = 8 for each treatment). The statistical significance was determined as compared to *Catharanthus* treated with 50 mM NaCl by p < 0.05 and p *<* 0.01 (*p < 0.05 and **p < 0.01*)*.

**Figure 8:**
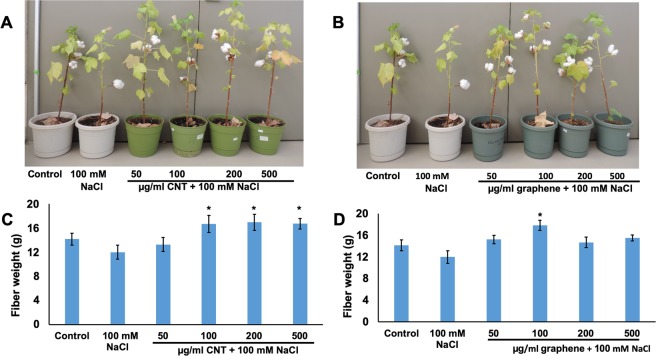
Long-term application of CBNs to salty soil reduced the toxic effects of salt and improved the growth and yield of cotton. The introduction of CBNs to salty soil positively affected the fiber yield of cotton cultivated in the soil supplemented with CNTs (**A**,**C**) and in soil supplemented with graphene (**B**,**D**) under imposed salt stress condition. Control cotton plants were grown in regular soil, NaCl treated cotton plants were grown in soil supplemented with 100 mM NaCl and CBNs exposed cotton plants were grown in salty soil (100 mM NaCl) supplemented with CNTs or graphene in a wide range of concentrations. The experiment was repeated twice (n = 8 for each treatment and for control) The statistical significance was determined as compared to cotton plants treated with 100 mM NaCl by p < 0.05 and p < 0.01 (*p < 0.05 and **p < 0.01*)*.

The greenhouse experiments revealed that cultivation of *Catharanthus* in salty soil led to significant reduction in overall plant growth including delayed flower production and changed plant architecture (Supplementary Fig. S7) as compared to *Catharanthus* cultivated in regular soil. For instance, cultivation of *Catharanthus* in NaCl supplemented soil resulted in a reduction of a total number of flower production by 64% as compared to untreated (control) *Catharanthus* (Supplementary Fig. S7C). However, the addition of nanomaterials (CNTs or graphene) to salty soil reduced the toxic effects of NaCl and improved several phenotypic traits including the early flower development (Supplementary Fig. S8) and total number of flowers as compared to *Catharanthus* plants exposed to NaCl mixed soil (Fig. 7). We observed that all the concentrations of CBNs tested were effective in reduction of toxic symptoms caused by NaCl and the activation of flower production in *Catharanthus* under NaCl mediated salt stress. For instance, the introduction of 100 µg/ml CNTs to salty soil (NaCl) led to an increase of total flower production by more than 200% as compared to *Catharanthus* plants cultivated in soil supplemented only with NaCl. Similarly, the introduction of 1000 µg/ml graphene to the salty soil led to the enhancement of flower production by two times as compared to *Catharanthus* plants cultivated in soil supplemented with only NaCl. Additionally, we also observed that the application of CBNs to salty soil significantly increased the total number of leaves produced by matured *Catharanthus* plants when compared to *Catharanthus* plant exposed to NaCl only (Supplementary Fig. S9). For example, the introduction of 50 µg/ml CNTs and 50 µg/ml graphene to *Catharanthus* cultivated salty soil led to an increase in total number of leaves by 65% and 41%, respectively, as compared to *Catharanthus* plants cultivated in soil supplemented with only NaCl. Similar results were recorded for NaCl exposed cotton plants by introduction of nanomaterials thorough watering with CBN solution for 4 weeks. Long-term application of CBNs reduced the toxic symptoms caused by NaCl and improved fiber yield under salt stress condition (Fig. 8). For instance, the introduction of 100–500 µg/ml CNTs and 200 µg/ml graphene to salty soil significantly increased cotton fiber biomass yield as compared to cotton plants cultivated in only NaCl treated the soil. Specifically, matured cotton plants exposed to 100 µg/ml CNTs and 100 µg/ml graphene increased the fiber biomass by 39% and 49%, respectively, as compared to cotton treated with NaCl only (Fig. 8).

### Effects of CBNs in the growth of *Catharanthus* under water deficit conditions

**Figure 9:**
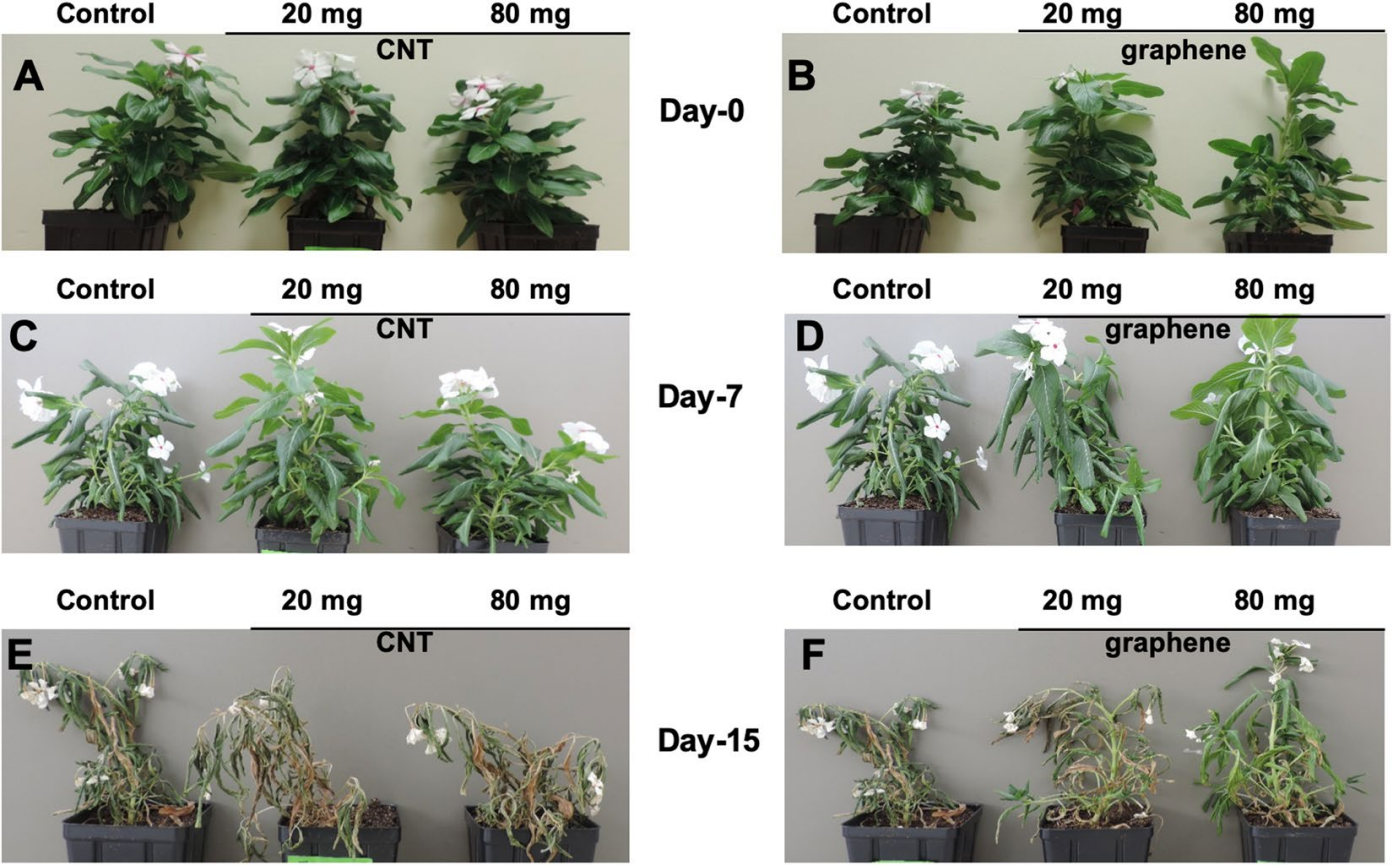
The phenotype of *Catharanthus* plants grown in conditions of water deficit stress in presence of CBNs. Effects of CNTs (**A**,**C**,**E**) and graphene (**B**,**D**,**F**) on the phenotype of *Catharanthus* at day-0 (**A**,**B**), day-7 (**C**,**D**) and day-15 (**E**,**F**) of water deficit stress. The final concentrations of CNTs and graphene were 20 mg and 80 mg per 400 g of soil mix. Delivery of CBNs to soil mix was achieved by the addition of CNTs or graphene solution to the soil for 4 weeks. The experiments were repeated twice (n = 10 for each treatment and for control).

**Figure 10:**
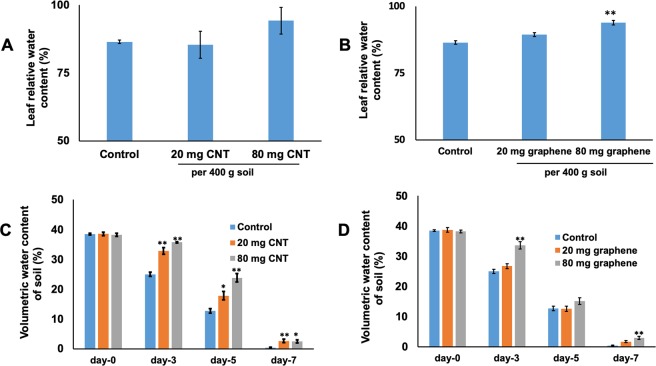
Effects of CBNs on leaf relative water content of *Catharanthus* cultivated at CNT -mixed soil (**A**) and graphene mixed soil (**B**) in conditions of more water deficit stress. Measurement of moisture content of *Catharanthus* cultivated soil mixed with CNTs (**C**) and graphene (**D**). The final concentrations of CNTs and graphene were 20 mg and 80 mg per 400 g of soil mix. Delivery of CNT to soil mix was achieved by the addition of CNT or graphene solution to the soil for 4 weeks. The experiments were repeated twice (n = 5 for (**A**,**B**) and n = 10 for (**C**,**D**) (*p < 0.05 and **p < 0.01).

In order to investigate the effects of CBNs in response to a water deficiency of ornamental species (*Catharanthus)*, 10- weeks-old CBN-exposed and control (untreated) *Catharanthus* plants were deprived of water for 2 weeks. After one week of drought stress, untreated *Catharanthus* plants (control) showed signs of water deficit stress as indicated by leaf wilting, while very slight stress symptoms were observed for *Catharanthus* plants previously treated with graphene or CNTs (Fig. 9C,D). After two weeks of drought stress, untreated *Catharanthus* plants were completely dried, whereas *Catharanthus* plants exposed to graphene showed the symptoms of leaf wilting but plants were not completely dried (Fig. 9E,F). The observed phenotypic difference between control and CBNs treated *Catharanthus* linked to doses of applied CBNs and intensity of water deficit stress of plants. We concluded that the application of CBNs can enhance the stress tolerance of *Catharanthus* against drought stress. Indeed, the exposure of mature *Catharanthus* to graphene or CNTs results in higher leaf relative water content as compared to leaves of untreated (control) *Catharanthus* plants (Fig. 10A,B). For example, the introduction of nanomaterials (80 mg CBNs per 400 g of soil) significantly increased the *Catharanthus* leaf relative water content. Moreover, measurement of the volumetric water content of pot soil used for plant cultivation revealed that the CBNs treated soil contained more moisture than the untreated soil at day 3, day 5, and day 7 of imposed drought stress (Fig. 10C,D). This observation clearly indicates that when CBNs is mixed with the soil, the soil moisture content will be maintained for longer period of time.

### Quantification of multi-walled CNTs translocated into leaves and flowers of CNT-exposed *Catharanthus* by microwave-induced heating technique (MIH) technique

The amount of CNTs absorbed by CNT-exposed *Catharanthus* was determined by using the MIH technique developed by Irin *et al*.^28^. The leaves and flowers collected from fully matured *Catharanthus* cultivated in regular soil, soil supplemented with CNTs, and soil supplemented with both NaCl and CNTs were used for the quantification of CNTs. The calibration curves for the detection of CNTs in the exposed *Catharanthus* organs are given in Fig. S10. The leaves and flowers of *Catharanthus* not exposed to CNTs were used as controls.

During the microwave exposure, CNT-exposed organs heated up much more than the control due to increased absorption of microwaves by CNTs in the organs as shown in Table 1. The temperature differences between the controls and the CNT-exposed organs were used to determine CNT content from the calibration curves. MIH analysis revealed that *Catharanthus* organs absorbed a relatively small amount of CNTs from both CNT-supplemented soil and CNT/NaCl-supplemented soil (Table 1). Specifically, matured *Catharanthus* cultivated in CNT supplemented soil (200 µg/ml) accumulated on average 0.014 ± 0.003 μg of CNTs per mg of dry leaves and 0.010 ± 0.002 μg of CNTs per mg of dry flowers. It was also determined that *Catharanthus* plants cultivated in soil-mix treated with CNTs (200 µg/ml) and NaCl (50 mM) accumulated similar amounts of CNTs as shown by leaves having 0.012 ± 0.003 µg of CNTs per mg of dry leaves and flowers having 0.009 ± 0.003 µg of CNTs per mg of dry flowers.

**Table 1:** Temperature differences observed during microwave exposure and the corresponding CNT contents inside the matured leaves and flowers of Catharanthus cultivated in CNT-supplemented soil under normal conditions as well as in the presence of NaCl.

Sample	Power, time	T_i_ (°C)	T_f_ (°C)	ΔT (°C)	μg CNT/mg sample	Avg. & STD
*Catharanthus* cultivated in regular soil mix (**leaves**)	30 W, 10 s	22	61	39	No CNTs	—
		22	61	39		
		22	59	37		
	50 W, 6 s	22	61	39		
		22	61	39		
		22	58	36		
*Catharanthus* cultivated in soil mix supplemented with 200 µg/ml CNTs solution (**leaves**)	30 W, 10 s	22	81	59	0.011	Avg = 0.014 STD = ±0.003
		22	82	60	0.011	
		22	84	62	0.012	
	50 W, 6 s	22	90	68	0.013	
		22	104	82	0.019	
		22	97	75	0.016	
*Catharanthus* cultivated in soil mix supplemented with 200 µg/ml CNTs solution and 50 mM NaCl (**leaves**)	30 W, 10 s	22	81	59	0.011	Avg = 0.012 STD = ±0.003
		22	77	55	0.009	
		22	81	59	0.011	
	50 W, 6 s	22	98	76	0.016	
		22	96	74	0.015	
		22	91	69	0.013	
*Catharanthus* cultivated in regular soil mix (**flowers**)	30 W, 10 s	21	70	49	No CNTs	—
		22	71	49		
		22	71	49		
	50 W, 6 s	22	75	53		
		22	76	54		
		22	77	55		
*Catharanthus* cultivated in soil mix supplemented with 200 µg/ml CNTs solution (**flowers**)	30 W, 10 s	22	87	65	0.009	Avg = 0.010 STD = ±0.002
		21	93	72	0.012	
		22	88	66	0.009	
	50 W, 6 s	22	104	82	0.012	
		22	99	77	0.010	
		21	102	81	0.011	
*Catharanthus* cultivated in soil mix supplemented with 200 µg/ml CNTs solution and 50 mM NaCl (**flowers**)	30 W, 10 s	21	81	60	0.006	Avg = 0.009 STD = ±0.003
		22	82	60	0.006	
		22	81	59	0.005	
	50 W, 6 s	22	103	81	0.011	
		22	104	82	0.012	
		22	107	85	0.013	

## Discussion

“Industrial crop” refers to the managed production of biological materials for the establishment of commercial non-food products^29^. Plants are used for the production of flowers or fiber bolls which are among the most valuable industrial species. Thus, the global revenue from ornamental horticulture (floriculture) is projected to supersede the valuation of US $43.2 billion, registering a robust annual growth rate of 7%, in 2019. The early development of flowers and fruits, as well as an increase in the number of produced flowers, are common desired traits of commercially valuable crops^30–32^. It is well known that ideal ornamental crops should produce high-quality flowers and leaf characters including aesthetic features, pigmentation, and plant architecture such as compact shape and enhanced branching^32^. Similarly, important characteristics of cotton are high-quality cotton boll production, early fiber initiation, enhanced number of fiber bolls and higher fiber yield^23,27^.

Our data indicate that nanotechnological approach can be a helpful approach for improving desirable traits of industrial crops rather than using genetic manipulations or application of harsh chemicals. We found that the application of two different types of CBNs (CNTs, graphene) to growth medium (agar or soil) improved several desirable traits of ornamental plants and cotton such as enhanced seed germination, early flower production, increased flower bud number, and improved tolerance to salt and water deficit stress. Thus, the introduction of CBNs to soil dramatically affected flower production and yield of ornamental plant *Catharanthus*. For example, *Catharanthus* plants cultivated in CNT and graphene mixed soil increased the total number of flowers by 37% and 58% respectively. Such an increase is quite significant and can be very beneficial for floriculture industry. Although long-term introduction of CBNs in the soil led to early flower production in cotton plants, we did not observe a significant increase in the fiber bolls in CBN-exposed cotton plants (Supplementary Fig. S2). However, it is possible that specific physiology of cotton plays an important role in the particular response of the reproductive system to CBNs exposure. The germination rate of seeds of both tested plant species was increased as a result of exposure to CBNs (CNTs, graphene) (Fig. 2). During plant development stage, the addition of CBNs to the growth medium resulted in early seedling development in cotton and *Catharanthus* with improved root and shoot growth as well as total biomass yield of young plants of both plant species (Fig. 3). The obtained results are in good agreement with our previous work that demonstrated the potential of CBNs in the enhancement of germination, development, and growth of model species (tomato)^11,13^, food crops (corn, soybean, and barley)^14^, and bioenergy species (sorghum and switchgrass)^15^.

Environmental stress is harmful to the horticultural industry since it reduces the germination rate and production of flowers drastically^20,21^. A significant reduction in the yield of a wide range of horticultural crops is being reported for one-third of irrigated land globally due to salt stress^22,23,33,34^. For example, irrigation of salty water to ornamental plants decreases plant height and lateral branches, and delay flower bud formation in *Tagetes patula* and *Ageratum mexicanum*^24^. Similarly, it was also documented that salt stress significantly affected cotton productivity by reducing the plant growth, biomass, and eventually hampered cotton bolls, as well as fiber yield^23^. Here, we demonstrated that application CBNs to soil may enhance plant tolerance to water deficit stress and salt stress (Figs. 7, 8). The continuous exposure of CNTs or graphene increased the total flower production by two times in *Catharanthus* under salt stress conditions. Similarly, matured cotton plants exposed to 100 µg/ml of CNT or graphene increased the fiber biomass by 39% and 49%, respectively. Our findings could be helpful for the improvement of plant growth in an arid and semi-arid region where salt stress is dominant. Therefore, the applications of CBNs to improve the plant yield along with plant desired traits is a profitable approach in the context of soil salinization.

The exact mechanism of the positive effects of CBNs on seed germination and plant growth is not fully clear and should be investigated in the future. However, we can suggest that improvement of salt and drought tolerance in CBN-treated plants can be linked with the impact of CBNs on genes/proteins involved in plant-water relationships. Previously, we showed that introduction of CNTs in plant growth medium led to the activation of expression of different types of water-channel genes (aquaporins) in a number of organs (seeds, roots, leaves) of a wide range of plant species^8,11,12,14,15^. Recently, Wang *et al*. (2019) proved that drought and salt tolerance can be significantly improved by overexpression of jojoba aquaporin gene in *Arabidopsis*^35^. Similarly, bamboo aquaporin confers drought and salinity tolerance in transgenic *Arabidopsis*^36^. It is logical to suggest that the observed positive effects of CBNs on salt and drought tolerance of cotton and *Catharanthus* can also be associated with overexpression of aquaporins caused by the application of CBNs. However, other possible mechanisms of influence of CBNs on plant stress tolerance should not be excluded. Recently, we demonstrated that CBNs can interact with toxic ions and reduce environmental toxicity. For example, it was shown that CNTs can physically interact with positively charged sodium ions and absorb toxic ions from the saline solution^15^. It was also noticed that transported nanomaterials inside the plants can bind with water molecules for a potential intracellular depot for the adjustment of osmotic stress^37^. For example, fullerene has binding capacity to large amounts of water and acts as a compatible osmolyte, which leads to the additional intracellular supply of water during osmotic stress in sugar beets^38,39^. Other nanomaterials can also act as a slow-release source of water. For example, hydrogel, nano zeolite, nanomaterials, and nanoclays have been successfully reported to enhance the water holding capacity of the soil and play a role in monitoring soil quality and plant health^40^. Based on the analysis of our data, we can suggest that a number of mechanisms may contribute to observed positive effects of CBNs on plant stress tolerance against environmental factors including interaction of CBN with harmful ions and influence of CBNs on plant stress signaling at molecular level.

Here, we confirmed the absorption of very low doses of CBNs by leaves and flowers of CNT-exposed *Catharanthus* plants using the microwave-heating method (MIH) (Table 1). However, since industrial plants are not used as a food source, risks associated with the transfer of CBNs residues located in flowers into the food chain can be less visible compared to food crops. At the same time, we cannot exclude that industrial plants contaminated with CBNs may be consumed by the animals and still find their way into the food chain. Previously, we have investigated possible risks associated with human/animal consumption of plant organs contaminated with CBNs using number of *in vitro* models^41^. We demonstrated that the amount of CNTs accumulated inside tomato fruits from CNT-exposed plants was not sufficient to initiate any change in transepithelial resistance and gene expression of model human intestinal epithelial cells (T-84)^41^. Additionally, we have reported that extracts from CNT-contaminated tomato fruits had no visible effect on human intestinal microbiota that was proved by 16s RNA sequencing^41^. Besides studies related to risk assessment of plants exposed to CBNs on human/animal health, environmental risks assessment should also be performed. Since CBNs used for regulation of plant growth and development will be delivered to the soil, it may affect soil microbiota. We have previously reported that the diversity and richness of soil microbial communities are not changed by application of CNTs used as plant growth regulators^16^. However, in order to prove safety of technologies associated with the use of CBNs in plant agriculture, more comprehensive risk assessment investigations have to be performed and reported.

## Conclusion

This work demonstrated the strong potential of CBNs as regulators of productivity and stress response of non-food plant species cultivated for flower/fiber boll consumption. The introduction of CBNs to soil dramatically affected the flower production and yield of ornamental plant *Catharanthus*. For instance, *Catharanthus* plants exposed to CNTs and graphene increased the total number of flowers by 37% and 58%, respectively. Such an improvement is quite significant and can be very beneficial for the floricultural industry. Both tested CBNs (CNTs, graphene) not only activated the reproductive system of cotton and *Catharanthus* beyond natural levels but also reversed toxicity caused by the two types of osmotic stresses: drought stress and salt stress. Addition of CBNs to growth medium helped both tested plant species to withstand environmental stress and produce flowers or fiber bolls at the level of plants that were not exposed to stress. The results of this work offers a promising nanotechnological approach for effective horticultural practices in the challenging conditions of climate change. Additionally, this study provides a new technological advancement for effective cultivation of flower-producing plants for space exploration since ornamental plants can provide a pleasant environment to astronauts, which leads to positive psychological effect^42^.

## Material and Methods

### Materials

Cotton seeds (MRC 270) were obtained from MRC Seeds (www.mrcseeds.com) Houston, TX, USA and *Catharanthus* seeds were obtained from JPK Seeds Company, CA, USA. Graphene nanoplatelets (<3 layers; lateral dimensions 1–2 µm) and multi-walled CNT-COOH (OD 13–18 nm; length 1–12 µm) were received from Cheap Tubes (Brattleboro, VT). Characterization of used CBNs by TEM and Raman spectroscopy was performed early and presented in previously published manuscript^12^. The dispersion of CBNs was performed using a Q Sonica, LLC (Newtown, CT) sonicator followed by three times autoclavation to remove endotoxins as reported previously^43^.

### Media preparation, seed germination, and development of seedlings of *Catharanthus* and cotton *in vitro*

To test the effects of nanomaterials on seed germination and growth of non-food crops (*Catharanthus*, cotton) under ordinary conditions, Murashige and Skoog medium (MS) was supplemented with two different concentrations of CBNs (50 µg/ml and 200 µg/ml of graphene or CNTs). To study the influence of CBNs on plants exposed to salt stress conditions, MS medium was supplemented with NaCl (50 mM NaCl for *Catharanthus* and 100 mM NaCl for cotton) and used and stress control medium. Other MS medium was supplemented with NaCl (50 mM NaCl for *Catharanthus* and 100 mM NaCl for cotton) and different concentration of CBNs (50 µg/ml, 100 µg/ml, 200 µg/ml, 500 µg/ml, 1000 µg/ml). The sterilization of *Catharanthus* seeds was performed by treating seeds with 70% ethanol followed by continuous vortexed with 20% bleach for 15 minutes. Similarly, cotton seeds were sterilized by treating with 70% ethanol followed by treatment with 3% hydrogen peroxide for 7 hours. Eventually, seeds of *Catharanthus* and cotton were washed with sterile water for nine times in aseptic conditions. The sterilized seeds of tested crops were placed in the control medium (medium without CBNs), medium supplemented with CBNs only, medium supplemented with NaCl, and medium supplemented with NaCl in presence of a different concentration of CBNs. For the *in vitro* experiments (seed germination, seedling growth), growth chamber was maintained at 25 °C with light intensity of 105 umol/s m^2^ for 12-h photoperiod. The percentage of seed germination was calculated for both tested species. The control and CBNs exposed seeds were further allowed for seedling development at both the normal and salt stress conditions. Phenotypic assessment of young seedlings was performed on day 28 and day 7 for *Catharanthu*s and cotton respectively.

### The growth and development of mature *Catharanthus* and cotton in greenhouse

To study the long-term effects of CBNs on growth and developments of selected crops (*Catharanthus*, cotton), young seedlings exposed to CNTs or graphene and control (unexposed) seedlings were grown in the greenhouse at the University of Arkansas at Little Rock, AR, USA. The conditions of the greenhouse experiments were maintained under a 16-h photoperiod at 30/25 °C (day/night) throughout the duration of the experiment. The 100 ml of CBNs (50 µg/ml and 200 µg/ml CNTs or graphene) were applied once a week for four consecutive weeks to the soil around root systems to 4 weeks old *Catharanthus* and 2 weeks old cotton plants. For the control plants, 100 ml of deionized water was applied. During the whole experimental period, an equal amount of deionized water was used for irrigation of CBNs treated as well as all control plants. Phenotypic measurements of mature plants were performed at 20 week-old *Catharanthus* plants and 23 week-old cotton plants.

Similarly, to investigate the long-term effects of CBNs on *Catharanthus*, and cotton exposed to salt stress conditions, eight young seedlings from each control, NaCl treated, and seedlings exposed to NaCl with graphene or CNTs as indicated above were transferred to the soil system. The 100 ml solution of graphene or CNTs (50, 100, 200, 500 and 1000 µg/ml) supplemented with NaCl was added to 4 week-old *Catharanthus* and 2 week-old cotton plants once a week for four consecutive weeks in the soil around the root system. For control plants, 100 ml of deionized water was applied whereas for salt-treated plants 100 ml of NaCl was solution supplied to plants once a week for four consecutive weeks. The equal amount of pure water was used for regular irrigation of control and CBN-treated plants on a daily base. The phenotypic analysis was done at 23 weeks old cotton and 25 weeks old *Catharanthus* cultivated in the greenhouse. To investigate the effects of CBNs on *Catharanthus* growth under water deficit stress, 10 week old untreated *Catharanthus* plants and *Catharanthus* exposed to graphene or CNTs as described previously were deprived of water for 2 weeks in the greenhouse conditions. After the supply of CBNs to the soil as 50 µg/ml and 200 µg/ml solutions, the final amount of CBNs in pots before water deficit stress experiment achieved 20 mg CBNs per 400 g soil and 80 mg CBNs per 400 g soil. The soil moisture level was quantified by measuring the volumetric water content in regular soil (no treatment) and soil pots supplemented with CBNs by using the decagon moisture sensor probe device (5TM soil moisture & temperature sensor) at day 0, day 3 day 5 and day 7 of drought stress. The changes in phenotypes of control and *Catharanthus* exposed to CBNs were observed closely for two weeks. To measure the leaf relative water content, five leaves were selected from each control *Catharanthus plant*, and plants grown in pots supplemented with CNTs or graphene. The leaf relative water content was calculated by measuring the fresh weight, turgid weight and dry weight of leaves^44^.

### Statistical analysis for experiments

To test the statistical significance for seed germination, plant growth and flower production/fiber boll production between control and CBNs exposed tested crop species, VASSARSTAT (http://vassarstats.net/anova1u.html) was used to perform One-way ANOVA. All data are expressed in average values ± SE (standard error). Statistical significance was determined as compared to untreated plants by p < 0.05 and p < 0.01 (*p < 0.05 and **p < 0.01).

### Quantification of multi-walled CNTs inside exposed *Catharanthus* using microwave-induced heating (MIH) technique

The uptake and translocation of CNTs by *Catharanthus* flower and leaf tissues were quantified using the advanced analytical MIH technique as described previously^11,15,28^. Approximately 100 mg of dried flowers and leaves were exposed for a given time and power to microwaves (2.45 GHz) in a custom waveguide, and the temperature rise was correlated with local CNT content. Prior calibration curves for ∆T vs. CNT loading (after 30 W, 10-sec exposure, and 50 W, 6-sec exposure) were used and adjusted as appropriate for these samples; the intercept is computed from the response of the control samples for each plant organ matrix. The difference in the temperature rise of the CNT-exposed plant organs and the control was used to extract the CNT content from the calibration curve.

## Supplementary information

Supplementary Information

## Data Availability

The datasets generated during and/or analyzed during the current study are available from the corresponding author on reasonable request.
