# Supplementary material for: Improvement of Commercially Valuable Traits of Industrial Crops by Application of Carbon-based Nanomaterials
Source: Sci Rep. 2019 Dec 18;9:19358. doi: 10.1038/s41598-019-55903-3 (PMC6920410; doi:10.1038/s41598-019-55903-3)
Supplement: Supplementary file 1 — Supplementary Information [file 41598_2019_55903_MOESM1_ESM.docx]

**Improvement of Commercially Valuable Traits of Industrial Crops by Application of Carbon-based Nanomaterials**

**Kamal Pandey^1^, Muhammad Anas^2^, Victoria K. Hicks^2^, Micah J. Green^2^, Mariya V. Khodakovskaya^1*^**

^1^Department of Biology, University of Arkansas at Little Rock, Little Rock, AR, 72204, USA

^2^Artie McFerrin Department of Chemical Engineering, Texas A&M University, College Station, TX, 77843, USA

*mvkhodakovsk@ualr.edu

**Figure S1.** Introduction of CNTs (A, C) and graphene (B, D) into soil led to the early flower production in *Catharantus* (A,B) and cotton (C, D) plants.


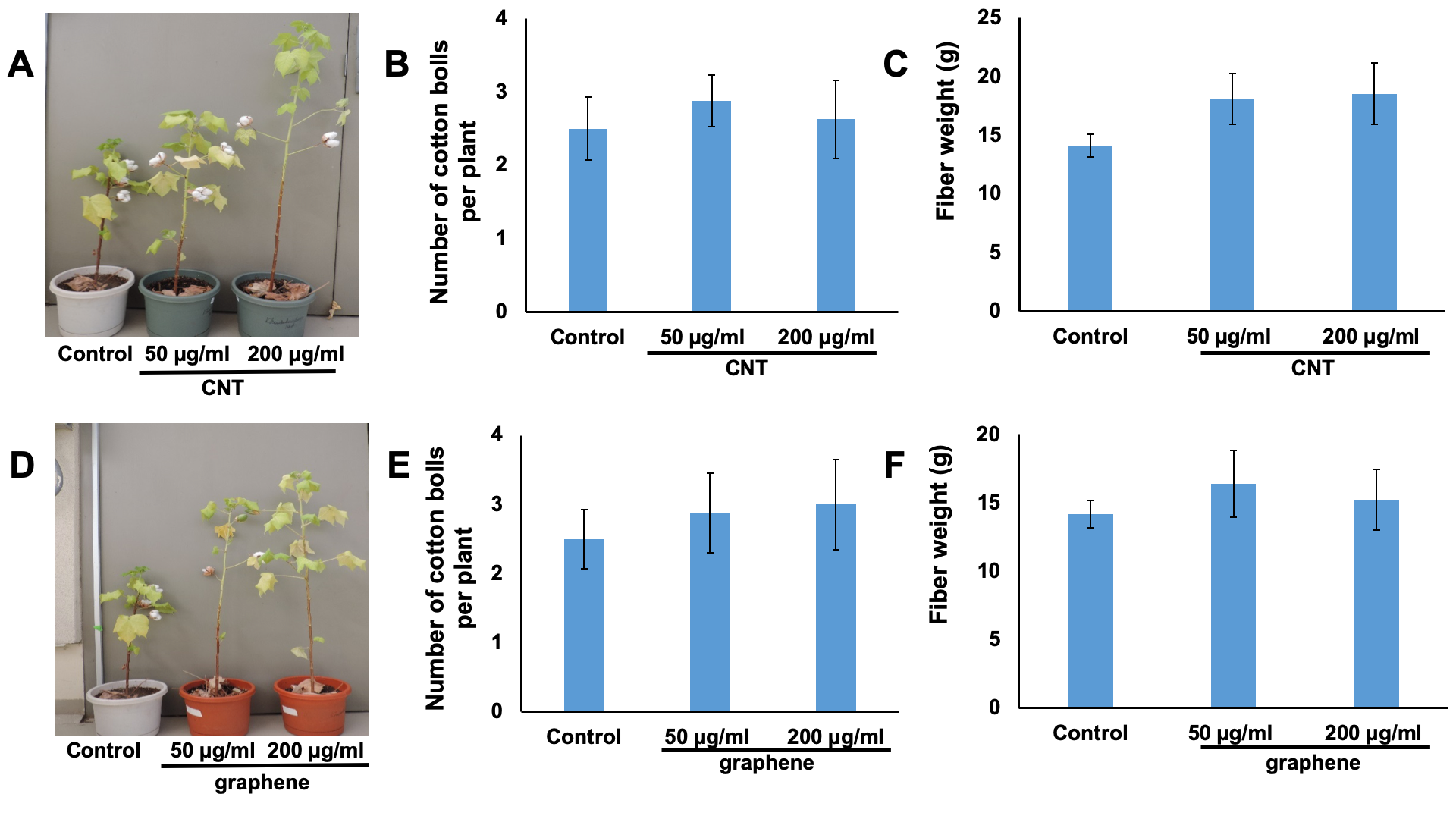


**Figure S2.** Effect of CNTs (A, B, C) and graphene (D, E, F) on fiber production of cotton cultivated in CBNs supplemented the soil. Phenotypes of 5-months old matured cotton (A, D). Effects of CBNs on fiber boll production (B, E) and fiber biomass yield (C, F) in matured cotton.

**
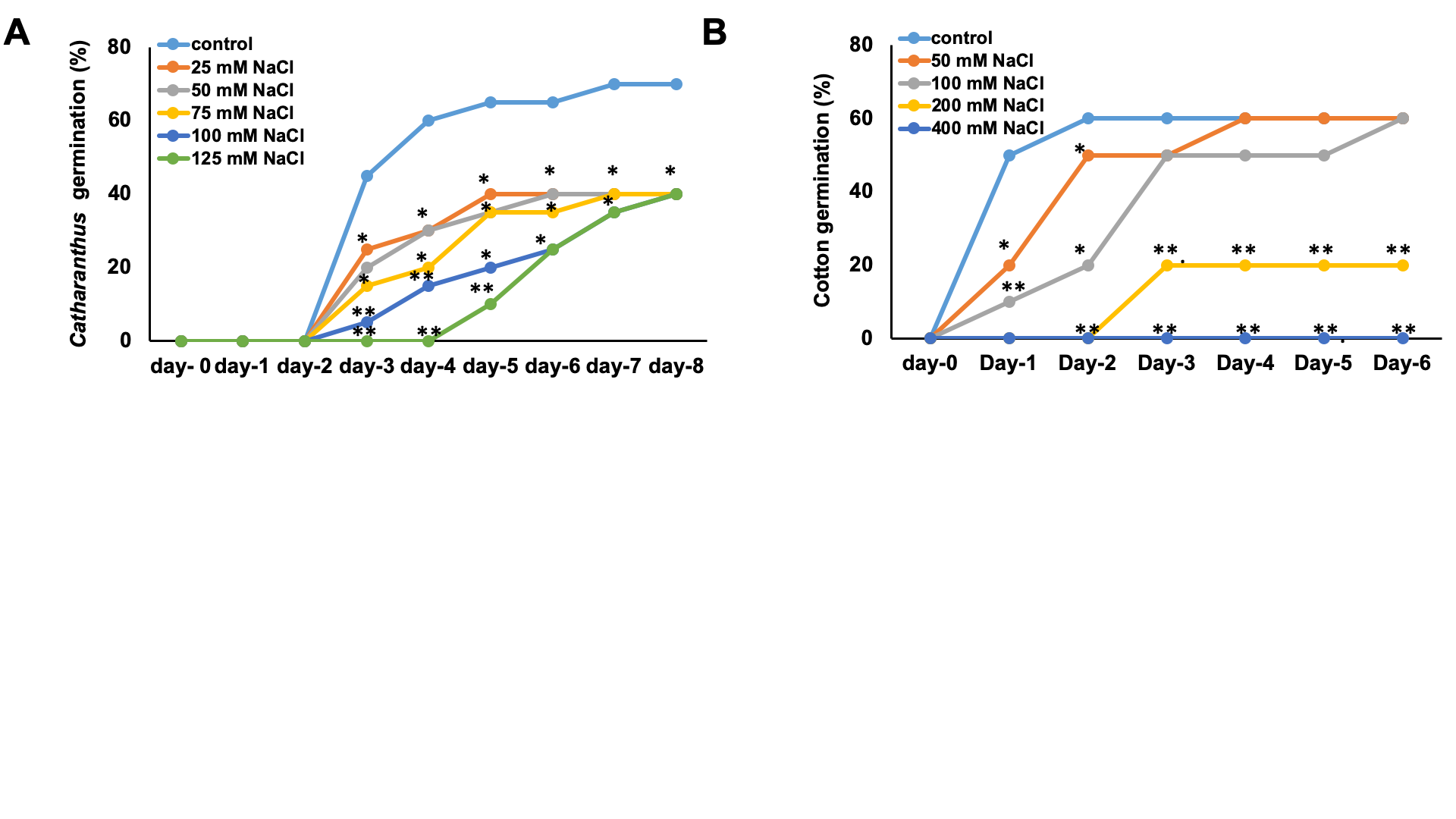
**

**Figure S3.** Effect of different concentration of NaCl on seed germination of *Catharanthus* (A) and cotton (B). The statistical significance is determined as compared to untreated (control) seeds (** p<0.01 and * p<0.05).

**Figure S4.** Effect of NaCl on the development of 4-weeks-old *Catharanthus* seedlings (A) and 1-week-old cotton seedlings (B).

**
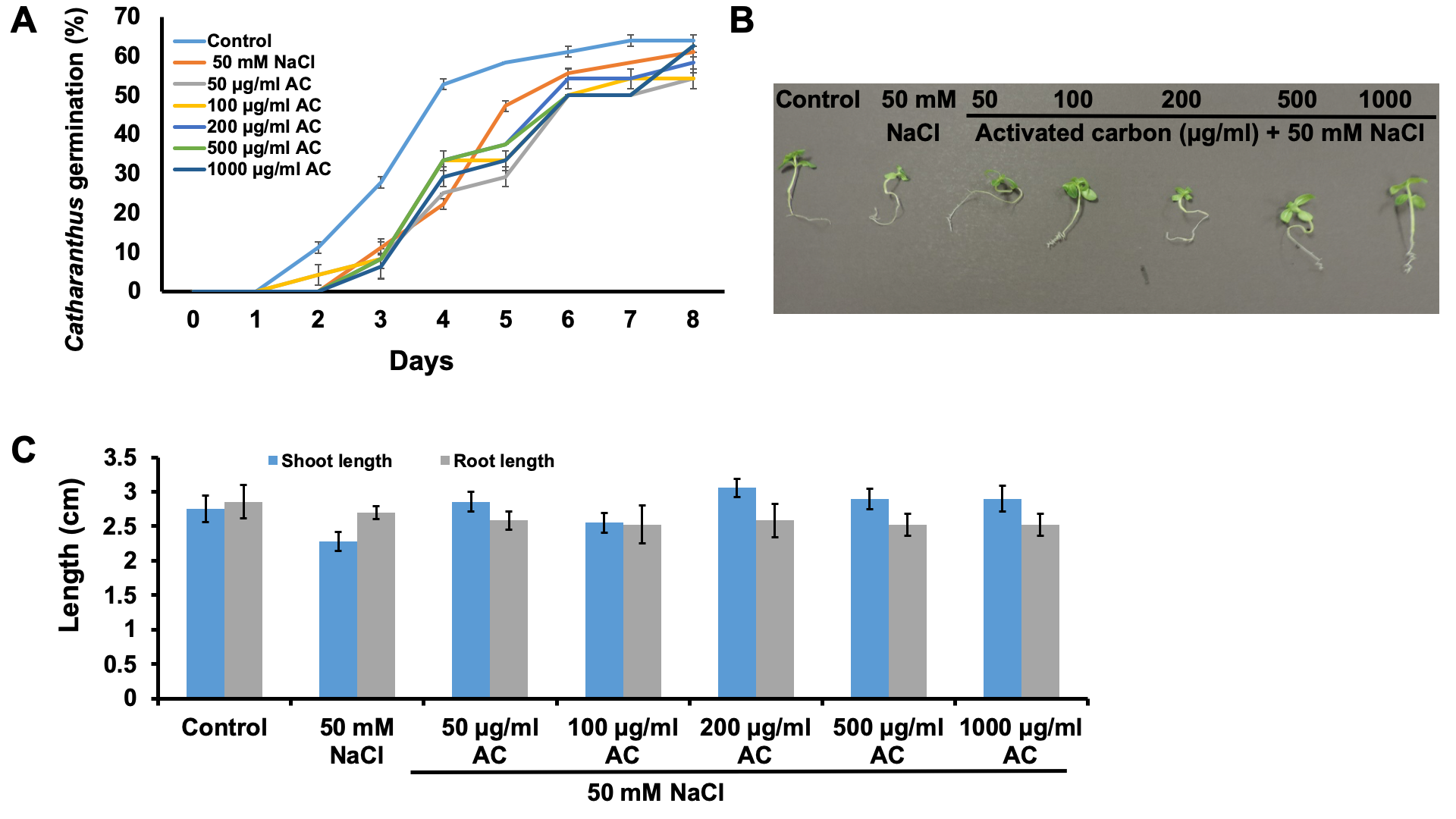
**

**Figure S5.** Effect of activated carbon on germination of *Catharanthus* seeds (A) and growth of 4-weeks-old *Catharanthus* seedlings (B, C) exposed to 50 mM NaCl. For control, seeds were placed on regular growth medium. For salt treatment, seeds were placed on growth medium supplemented with 50 mM NaCl. For treatment with activated carbon, seeds were placed on medium supplemented with 50 mM NaCl and different concentrations of activated carbon (50, 100, 200, 500, 1000 µg/ml).

**Figure S6.** Effect of CNTs (A) and graphene (B) on the biomass of shoots and roots produced by 4-weeks-old *Catharanthus* seedlings grown under salt stress. The statistical significance is determined as compared to *Catharanthus* treated with 50 mM NaCl (** p<0.01 and * p<0.05).

**Figure S7.** Reduction of growth and delay of flower development of *Catharanthus* in response to the application of 50 mM NaCl to the soil. Phenotypes of 12-weeks-old *Catharanthus* plants (A). Cultivation of *Catharanthus* in soil supplemented with NaCl resulted in delay of flower development (B) and reduction of total number of produced flowers (C). The statistical significance is determined as compared to untreated (control) (** p<0.01 and * p<0.05).

**Figure S8.** CNT (A) and graphene (B) reduced toxic effects of NaCl on flower production in *Catharanthus*. The effects of CNT(A) and graphene (B) on flower development in *Catharanthus* cultivated in soil supplemented with NaCl.


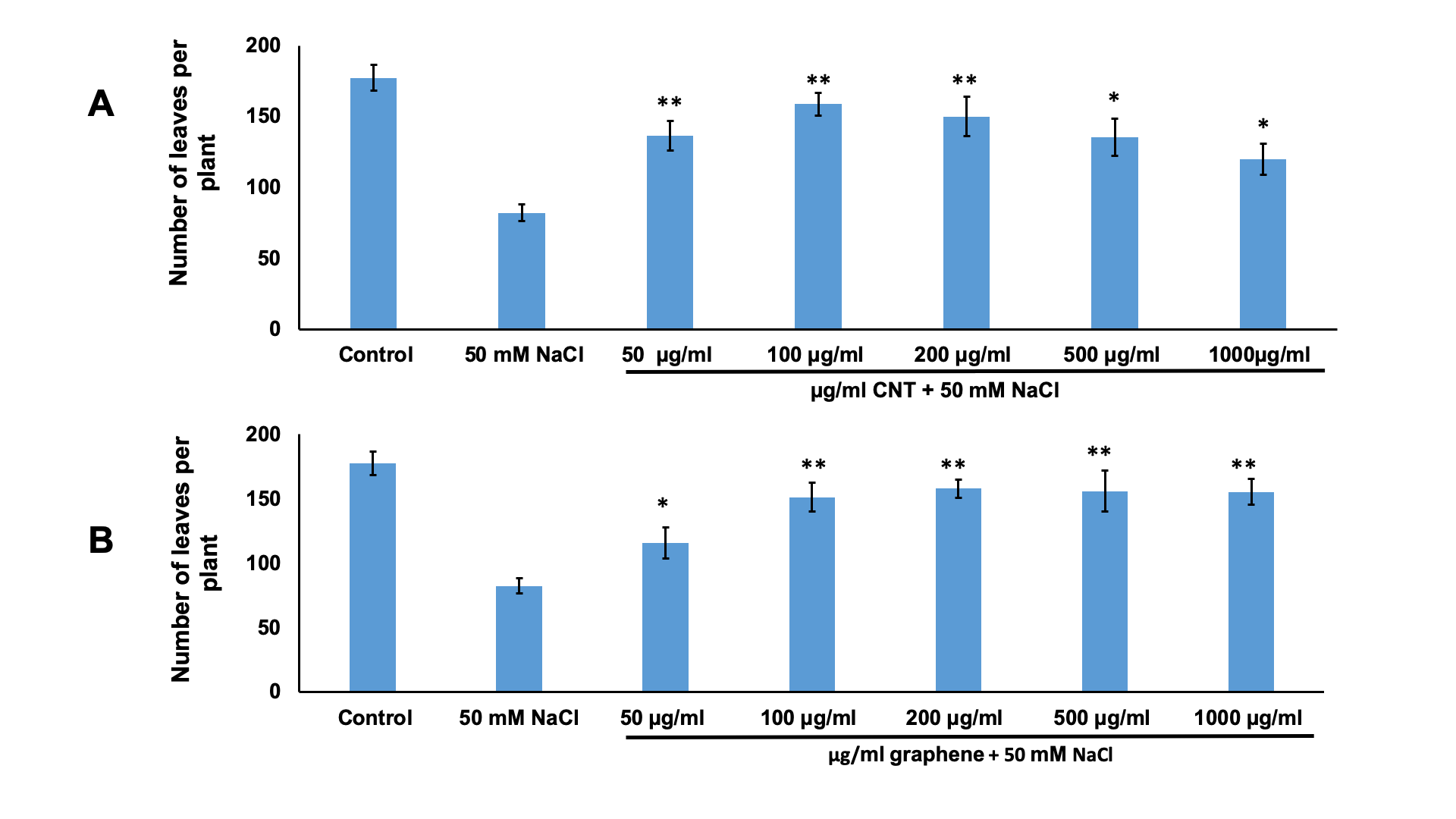


**Figure S9.** The effects of CNTs (A) and graphene (B) on a total number of leaves produced by *Catharanthus* under salt stress (50 mM NaCl). For control, plants were grown in regular soil only. For NaCl treatment, plants were grown in the soil supplemented with 50 mM NaCl. For treatment with CBNs, plants were grown in the soil supplemented with 50 mM NaCl and different concentrations of CNTs or graphene (50, 100, 200, 500, 1000 µg/ml). The statistical significance is determined as compared to *Catharanthus* treated with 50 mM NaCl (** p<0.01 and * p<0.05).


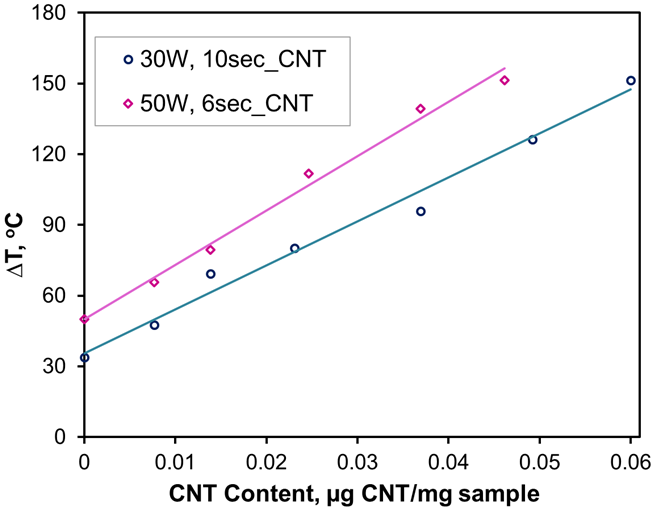


**Figure S10.** Calibration curves at two different microwave exposure conditions used for the detection of multi-walled CNTs in the leaves and flowers of CNT-exposed *Catharanthus.*

**Table S1:** Fresh biomass yield of 4 weeks old *Catharanthus* seedlings exposed to CNT or graphene

| **Treatment** | **Fresh shoot biomass (g)** | **Fresh root biomass (g)** |
| --- | --- | --- |
| Control | 0.0206 ± 0.0014 | 0.0017 ± 0.0002 |
| 50 µg/ml CNT | 0.0274 ±0.0038** | 0.0025 ± 0.0005** |
| 200 µg/ml CNT | 0.0241 ± .0018** | 0.0031 ± 0.0002** |
| 50 µg/ml graphene | 0.0271 ± 0.0008** | 0.0037 ± 0.0000** |
| 200 µg/ml graphene | 0.0316 ± 0.0008** | 0.0028 ± 0.0000** |

**Table S2:** Fresh biomass yield of 1 week old cotton seedlings exposed to CNT or graphene

| **Treatment** | **Fresh shoot biomass (g)** | **Fresh root biomass (g)** |
| --- | --- | --- |
| Control | 0.578 ± 0.080 | 0.11 ± 0.018 |
| 50 µg/ml CNT | 0.593 ± 0.080 | 0.151 ± 0.020* |
| 200 µg/ml CNT | 0.606 ± 0.030 | 0.153 ± 0.000* |
| 50 µg/ml graphene | 0.636 ± 0.080 | 0.141± 0.030* |
| 200 µg/ml graphene | 0.808 ± 0.050* | 0.176 ± 0.010* |

The statistical significance was determined as compared to control (untreated) seedlings (** p<0.01 and * p<0.05).
